# Fast and Accurate Amyloid Brain PET Quantification Without MRI Using Deep Neural Networks

**DOI:** 10.2967/jnumed.122.264414

**Published:** 2023-04

**Authors:** Seung Kwan Kang, Daewoon Kim, Seong A Shin, Yu Kyeong Kim, Hongyoon Choi, Jae Sung Lee

**Affiliations:** 1 Brightonix Imaging Inc., Seoul, Korea;; 2 Institute of Radiation Medicine, Medical Research Center, Seoul National University College of Medicine, Seoul, Korea;; 3 Interdisciplinary Program of Bioengineering, Seoul National University, Seoul, Korea;; 4 Artificial Intelligence Institute, Seoul National University, Seoul, Korea;; 5 Department of Nuclear Medicine, Seoul National University College of Medicine, Seoul, Korea; and; 6 Department of Nuclear Medicine, Seoul Metropolitan Government–Seoul National University Boramae Medical Center, Seoul, Korea

**Keywords:** amyloid PET, spatial normalization, deep learning, quantification

## Abstract

This paper proposes a novel method for automatic quantification of amyloid PET using deep learning–based spatial normalization (SN) of PET images, which does not require MRI or CT images of the same patient. The accuracy of the method was evaluated for 3 different amyloid PET radiotracers compared with MRI-parcellation–based PET quantification using FreeSurfer. **Methods:** A deep neural network model used for the SN of amyloid PET images was trained using 994 multicenter amyloid PET images (367 ^18^F-flutemetamol and 627 ^18^F-florbetaben) and the corresponding 3-dimensional MR images of subjects who had Alzheimer disease or mild cognitive impairment or were cognitively normal. For comparison, PET SN was also conducted using version 12 of the Statistical Parametric Mapping program (SPM-based SN). The accuracy of deep learning–based and SPM-based SN and SUV ratio quantification relative to the FreeSurfer-based estimation in individual brain spaces was evaluated using 148 other amyloid PET images (64 ^18^F-flutemetamol and 84 ^18^F-florbetaben). Additional external validation was performed using an unseen independent external dataset (30 ^18^F-flutemetamol, 67 ^18^F-florbetaben, and 39 ^18^F-florbetapir). **Results:** Quantification results using the proposed deep learning–based method showed stronger correlations with the FreeSurfer estimates than SPM-based SN using MRI did. For example, the slope, *y*-intercept, and *R*^2^ values between SPM and FreeSurfer for the global cortex were 0.869, 0.113, and 0.946, respectively. In contrast, the slope, *y*-intercept, and *R*^2^ values between the proposed deep learning–based method and FreeSurfer were 1.019, −0.016, and 0.986, respectively. The external validation study also demonstrated better performance for the proposed method without MR images than for SPM with MRI. In most brain regions, the proposed method outperformed SPM SN in terms of linear regression parameters and intraclass correlation coefficients. **Conclusion:** We evaluated a novel deep learning–based SN method that allows quantitative analysis of amyloid brain PET images without structural MRI. The quantification results using the proposed method showed a strong correlation with MRI-parcellation–based quantification using FreeSurfer for all clinical amyloid radiotracers. Therefore, the proposed method will be useful for investigating Alzheimer disease and related brain disorders using amyloid PET scans.

## Visual Abstract

**Figure:**
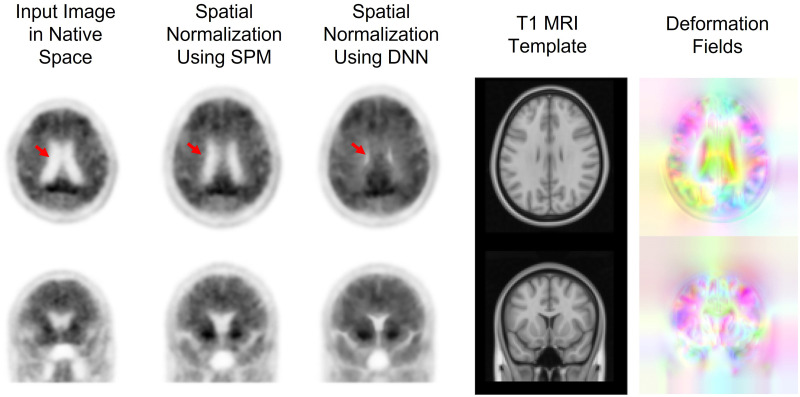

Because of the nature of brain diseases, the pathologic condition of the brain should be evaluated noninvasively. PET is a useful imaging tool for assessing the functional and molecular status of the brain (1,2). The application of brain PET imaging in the diagnosis and treatment of degenerative brain diseases is widely increasing (3–5). In Alzheimer disease (AD), the most common degenerative brain disease, brain deposition of fibrillar amyloid β-plaques is a neuropathologic hallmark for diagnosis. Therefore, amyloid PET has significantly contributed to the diagnosis and treatment of AD.

Visual assessment of PET images by nuclear medicine physicians or radiologists is the standard method for clinical neuroimaging interpretation. Nevertheless, quantitative and statistical analyses of PET images are widely used in brain disease research (1*,*^2^*,*^6^–^9^) because such analyses provide useful information for objective interpretation of the PET images of individual patients. The most prevalent method of quantitative image analysis is evaluating regional uptake of radiotracers by manually drawing a region of interest or volume of interest (VOI) on individual brain PET images. Another common method for brain PET image analysis is voxelwise statistical analysis, which is based on spatial normalization (SN) of images (10–12). Furthermore, brain PET SN allows the use of predefined VOIs, which are a suitable alternative to laborious and time-consuming manual VOI drawing (13–19).

Monoclonal antibodies such as aducanumab and donanemab are emerging as AD treatment drugs that target aggregated amyloid β to reduce its buildup in the brain (20*,*^21^). Therefore, the importance of quantification methods for amyloid brain PET images with high objectivity, accuracy, and reproducibility is increasing. Although voxelwise statistical analysis and predefined-VOI–based automated anatomic labeling are objective and efficient methods for amyloid brain PET image analysis, their reliability depends primarily on the accuracy of the SN procedure. However, accurate amyloid PET SN without the complementary use of anatomic images, such as MRI or CT, is technically challenging because of the large discrepancy in amyloid deposit patterns between cognitively normal and abnormal cases (22–24). Additionally, severe cerebral atrophy and hydrocephalus, which are frequently observed in older patients, complicate SN. Previously, we proposed 2 deep-learning–based amyloid PET SN methods that did not require matched MRI or CT data (25*,*^26^). In one of these approaches (25), we used a generative adversarial network to generate pseudo-MRI data from amyloid PET and applied spatial transformation parameters—obtained by performing SNs of pseudo-MR images on the MRI template—to amyloid PET images. In the second approach (26), we used deep neural networks (DNNs) to generate adaptive PET templates for individual amyloid PET images and performed SN of amyloid PET images using individual adaptive templates. Both approaches showed a strong correlation of regional SUV ratio (SUVR) relative to cerebellar activity with the matched MRI-based PET SN and outperformed the MRI-less SN with the average amyloid PET template. However, these methods have the following limitations: first, the process of generating a pseudo-MRI or adaptive template using DNNs and the SN process are separated. Second, we used the SN algorithm provided by the Statistical Parametric Mapping (SPM; Wellcome Centre for Human Neuroimaging) software, which iteratively applies image registration and segmentation algorithms (27). Therefore, the accuracy and speed of the entire SN pipeline depend on the SN performance and computation time of SPM. These limitations undermine the advantage of not requiring matched MRI for amyloid PET SN in both approaches.

Therefore, in this study, we developed a novel MRI-less amyloid PET SN method that allows 1-step generation of spatially normalized PET images using cascaded DNNs that estimate linear and nonlinear SN parameters from individual amyloid PET images. Furthermore, we evaluated the accuracy of the proposed method for 3 different amyloid PET radiotracers compared with MRI-parcellation–based PET quantification using FreeSurfer (28), which has shown a strong correlation with a manual-drawing method in cortical thickness and volume measurement (29–31) and in regional amyloid load estimation (32*,*^33^) but requires a significantly longer computation time (∼8 h).

## MATERIALS AND METHODS

### Datasets

To train and test the DNN model for PET SN, we used an open-access dataset provided by the National Information Society Agency (https://aihub.or.kr/). This internal dataset comprised pairs of multicenter amyloid PET scans (^18^F-florbetaben or ^18^F-flutemetamol) and structural T1-weighted 3-dimensional MRI scans of patients with AD or mild cognitive impairment and cognitively normal subjects. The image data were acquired from 6 university hospitals in South Korea. The demographic information and clinical diagnoses of the training and test sets are summarized in Table 1. A public institutional bioethics committee designated by the Ministry of Health and Welfare of South Korea approved the retrospective use of the scan data and waived the need for informed consent.

**TABLE 1.** Demographic and Clinical Diagnosis of Training and Test Datasets

			Sex		Diagnosis			Tracer	
Parameter	*n*	Age (y)	M	F	NC	MCI	AD	FMM	FBB
Training set	994	73.2 ± 5.6	318	676	200	543	251	367	627
Test set	148	74.8 ± 6.6	75	73	26	85	37	64	84

NC = cognitively normal control; MCI = mild cognitive impairment; FMM = ^18^F-flutemetamol; FBB = ^18^F-florbetaben.

Furthermore, the trained network was evaluated using an external dataset obtained from the Global Alzheimer Association Interactive Network (http://www.gaain.org/centiloid-project). The trained network was tested for 3 different Food and Drug Administration–approved amyloid tracers: ^18^F-florbetaben, ^18^F-flutemetamol, and ^18^F-florbetapir. Originally, this dataset, comprising young controls and elderly subjects, was acquired for the centiloid calibration of each tracer (34–36). The demographic information is summarized in Table 2.

**TABLE 2.** Demographic and Clinical Diagnosis of External Test Dataset

		Diagnosis	
Tracer	*n*	Young control	Elderly
^18^F-florbetaben	30	8	22
^18^F-flutemetamol	67	22	45
^18^F-florbetapir	39	12	27

Age and sex were anonymized.

### Network Model

**FIGURE 1.**
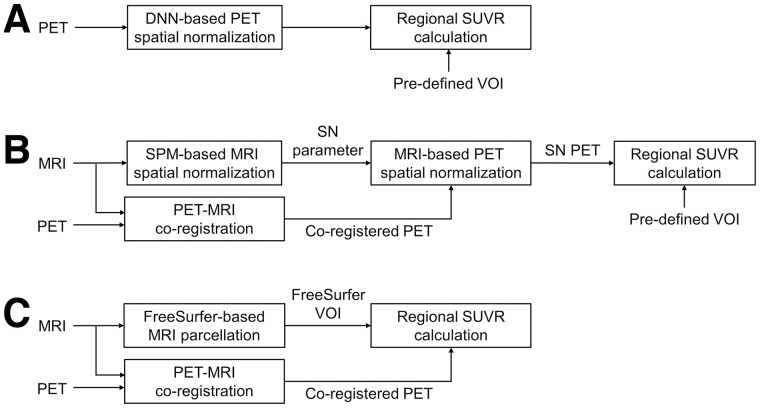
Three approaches used to estimate regional SUVR from amyloid PET images are compared in this study: DNN-based PET SN (A), PET/MRI coregistration and MRI-based PET SN using SPM (B), and PET/MRI coregistration and MRI parcellation using FreeSurfer (C).

The proposed DNN model, comprising cascaded U-nets (37*,*^38^), takes an affine-registered amyloid PET image as input and generates local displacement fields for nonlinear registration (Supplemental Fig. 1; supplemental materials are available at http://jnm.snmjournals.org). The generated displacement fields were then applied to the coregistered MR images in the training phase, and the cross-correlation loss between the spatially normalized MR images and the T1 template (individual Montreal Neurological Institute [MNI] 152) was minimized by error back propagation. Additionally, the gray matter segment of each MR image was used to improve the performance of the trained network and deformed using the same displacement fields as shown in Supplemental Figure 1. Dice loss was calculated between the deformed gray matter segment and the gray matter of the MNI 152 template, which was minimized along with the cross-correlation loss. On-the-fly data augmentation was applied when training the network model to prevent parameter overfitting. Spatially normalized PET images were not required in the training phase, and only PET images in individual spaces were used to create deformation fields. When the DNN model was trained, only PET images in an individual space were fed into the DNN model to generate SN images in the template space (Fig. 1A).

### Quantification of Amyloid Load

SN was conducted using the SPM program (version 12; https://www.fil.ion.ucl.ac.uk/spm) for comparison (Fig. 1B). Using the SPM program, PET and MRI pairs were coregistered, and the MR images were spatially normalized. MRI SN was performed using a unified segmentation method that applies tissue probability maps as deformable spatial priors for regularization of the nonlinear deformations (27). The PET images were then spatially normalized using the deformation fields estimated from the paired MRI.

Using the VOIs predefined in the template space, regional PET counts were extracted from spatially normalized images using DNN or SPM. The predefined VOIs were generated by applying automatic MRI parcellation using FreeSurfer software (version 7.1.0; Martinos Center for Biomedical Imaging) to the MNI template (39*,*^40^). The cortical and subcortical structures segmented and parcellated by FreeSurfer were grouped into 6 composite VOIs: global cerebral cortex, frontal lobe, posterior cingulate cortex and precuneus, lateral parietal, lateral temporal, and medial temporal. The counts of the VOIs were then divided by the counts of the cerebellar gray matter to calculate SUVR.

As a reference, SUVRs in individual brain spaces were estimated using T1-weighted 3-dimensional MR images and FreeSurfer (Fig. 1C). The results of the FreeSurfer segmentation of MR images were visually inspected by a neuroscience expert to ensure the quality of all datasets. About 10% of the datasets were excluded because of incomplete cortex segmentation or cessation of the FreeSurfer program. Cases of failure were higher in elderly subjects (young controls, 8.7%; elderly, 10.5%). Finally, the 6 composite VOIs were applied to the coregistered amyloid brain PET images to calculate SUVR. FreeSurfer SUVR estimated in individual space was regarded as ground truth because FreeSurfer and manual-drawing approaches achieved nearly identical estimates of amyloid load (32).

### Statistical Analysis

The correlation between SN-based approaches (DNN or SPM) and the FreeSurfer approach was evaluated using Pearson correlation. Furthermore, we performed a Bland–Altman analysis on the SUVR. Additionally, intraclass correlation coefficients were calculated to assess the consistency of the quantification results.

## RESULTS

**FIGURE 2.**
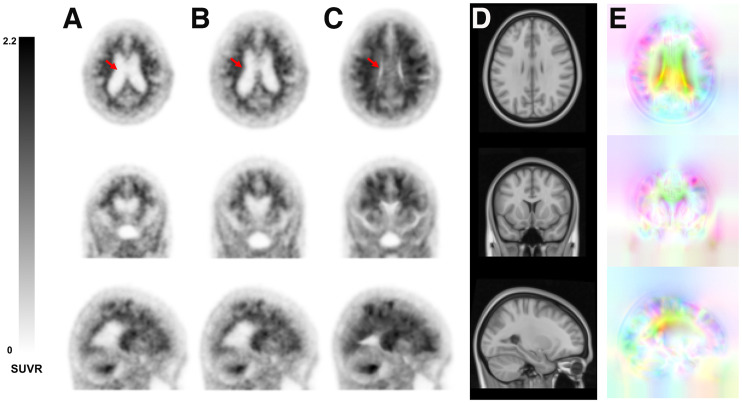
SN of ^18^F-florbetaben PET in amyloid-positive case: input image in individual space (A), MRI-based SN using SPM (B), PET SN using DNN (C), T1 MRI template (D), and estimated deformation fields using DNN (E). Red arrows indicate the enlarged ventricles, which are not properly deformed by SPM.

After network training, the proposed DNN method successfully generated displacement fields for SN and achieved accurate spatially normalized PET images, as shown in Figure 2 and Supplemental Figure 2. However, the SPM SN was not sufficiently accurate for patients with severe ventricular enlargement (Fig. 2; Supplemental Fig. 2); nonetheless, the ventricular enlargement did not degrade the performance of the proposed method. Figure 2 and Supplemental Figure 2 show a representative amyloid-positive case and an amyloid-negative case with a global SUVR of 1.889 (73-y-old woman; diagnosis, AD; tracer,^18^F-florbetaben) and 1.318 (80-y-old woman; cognitively normal; tracer,^18^F-florbetaben), respectively.

**FIGURE 3.**
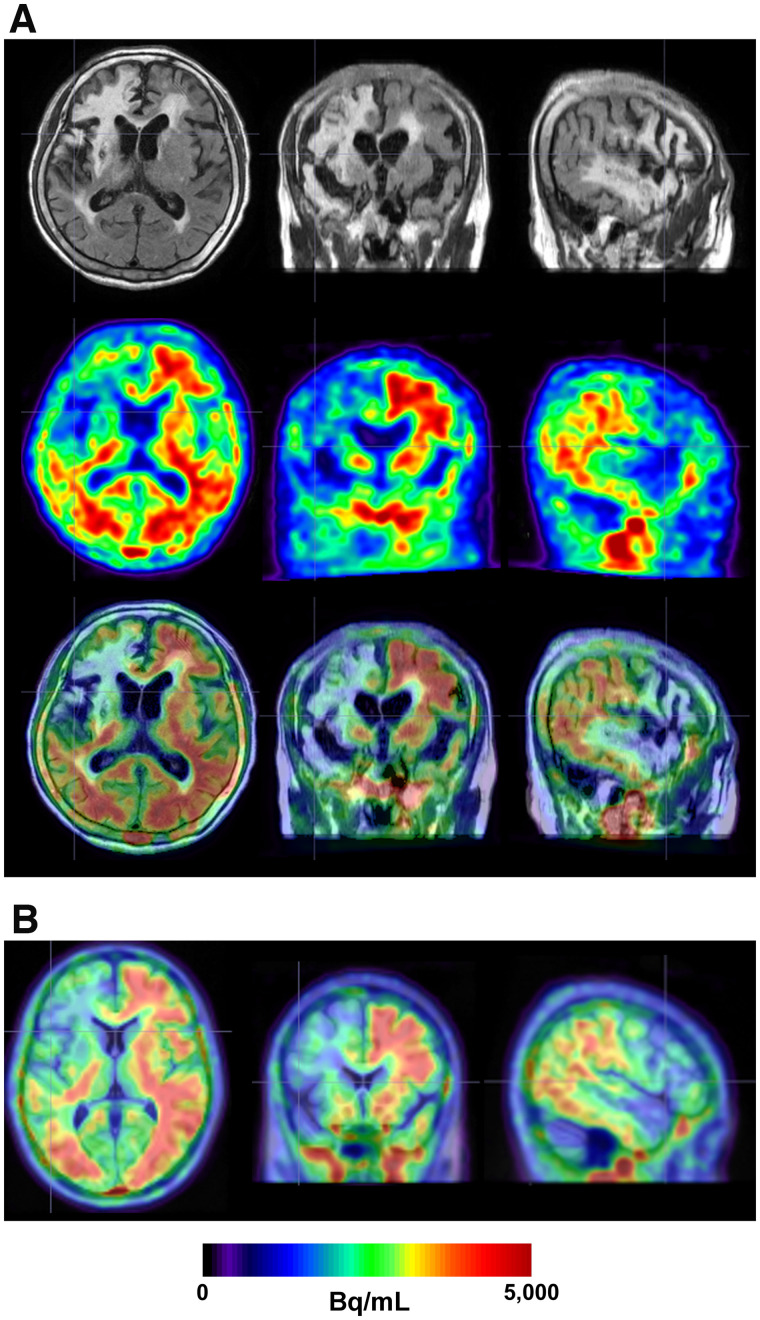
SN result of patient with chronic stroke lesion using proposed method. (A) Patient’s original FLAIR MRI (top), ^18^F-florbetaben (middle), and PET/MRI fusion (bottom). (B) SN PET overlaid on standard T1 MRI template.

The proposed DNN method is also robust in the SN of lesioned brains. Figure 3 and Supplemental Figure 3 show the SN result for a patient (84-y-old woman; tracer,^18^F-florbetaben) with a chronic stroke lesion using the proposed method, thereby enabling accurate SN with no shrinkage in lesion volume.

**FIGURE 4.**
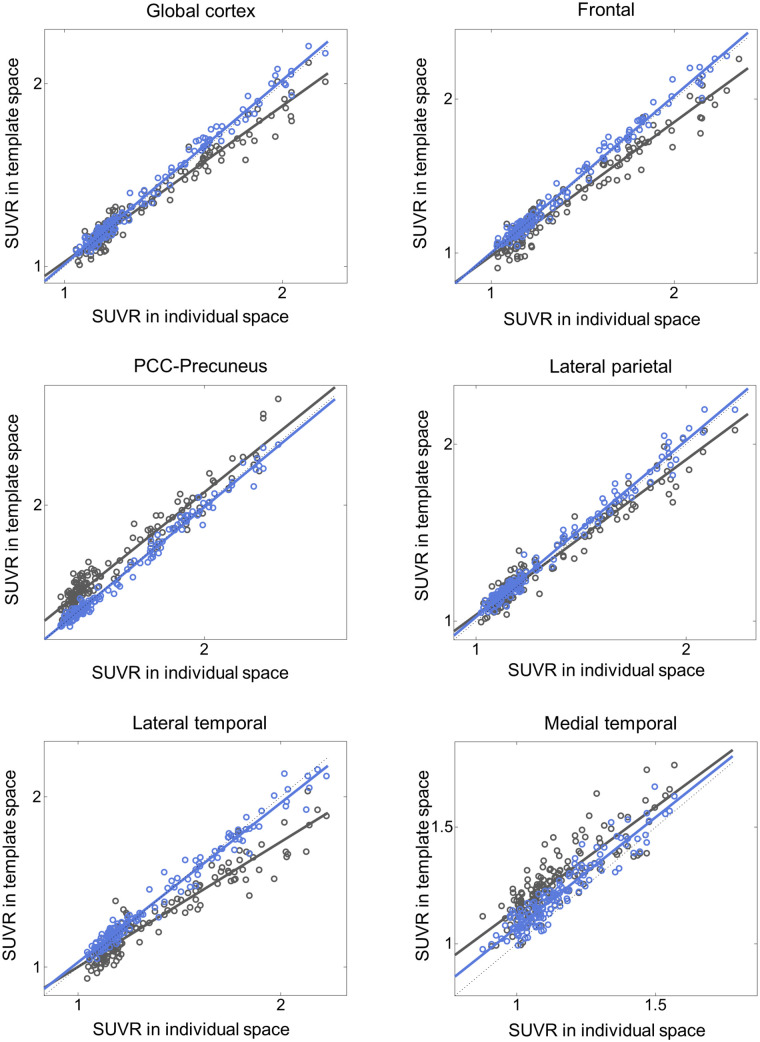
Internal validation: SUVR comparison in ^18^F-florbetaben and ^18^F-flutemetamol (*n* = 148). *x*-axis represents ground truth SUVR estimated in individual space using FreeSurfer VOI, whereas *y*-axis represents SUVR estimated in template space using coregistered MRI and SPM (black symbols and lines) or proposed DNN (blue symbols and lines). PCC = posterior cingulate cortex.

**FIGURE 5.**
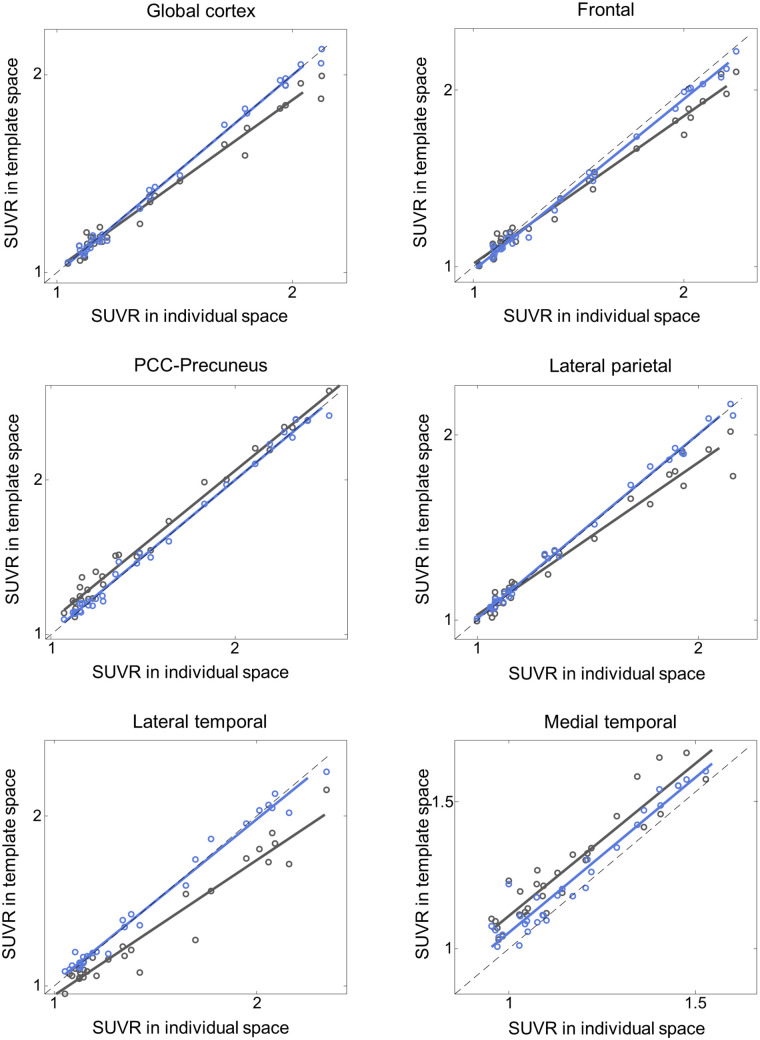
External validation: SUVR comparison in ^18^F-florbetaben (*n* = 30). *x*-axis represents ground truth SUVR estimated in individual space using FreeSurfer VOI, whereas *y*-axis represents SUVR estimated in template space using coregistered MRI and SPM (black symbols and lines) or proposed DNN (blue symbols and lines). PCC = posterior cingulate cortex.

**FIGURE 6.**
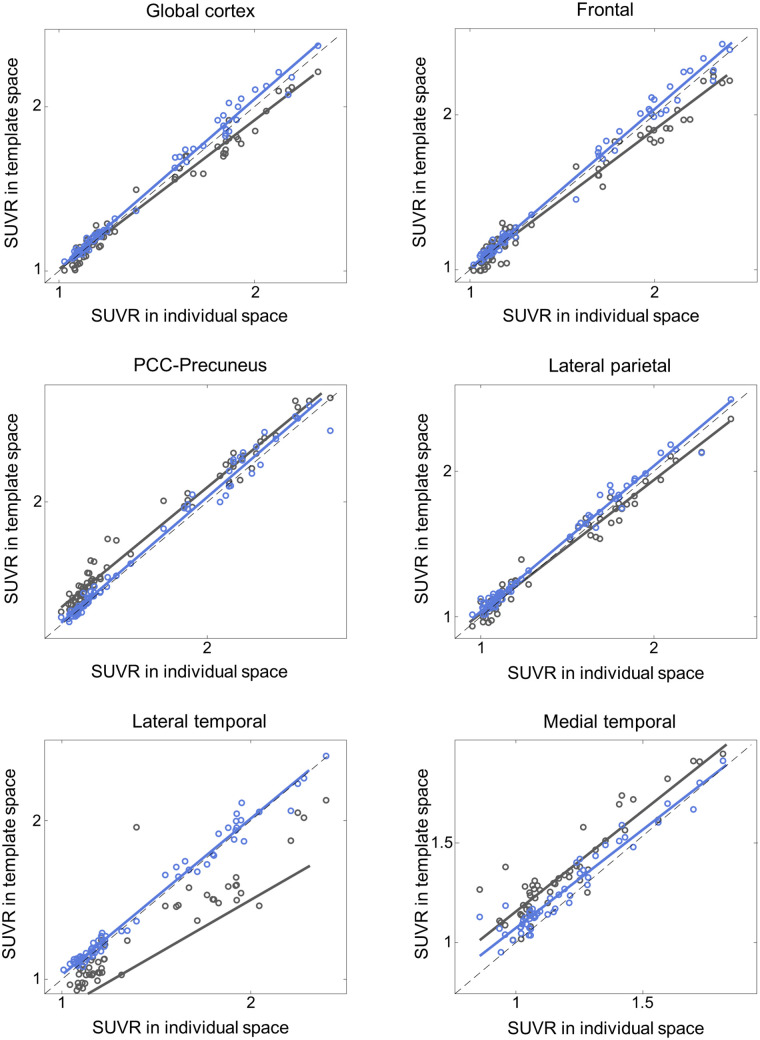
External validation: SUVR comparison in ^18^F-flutemetamol (*n* = 67). *x*-axis represents ground truth SUVR estimated in individual space using FreeSurfer VOI, whereas *y*-axis represents SUVR estimated in template space using coregistered MRI and SPM (black symbols and lines) or proposed DNN (blue symbols and lines). PCC = posterior cingulate cortex.

**FIGURE 7.**
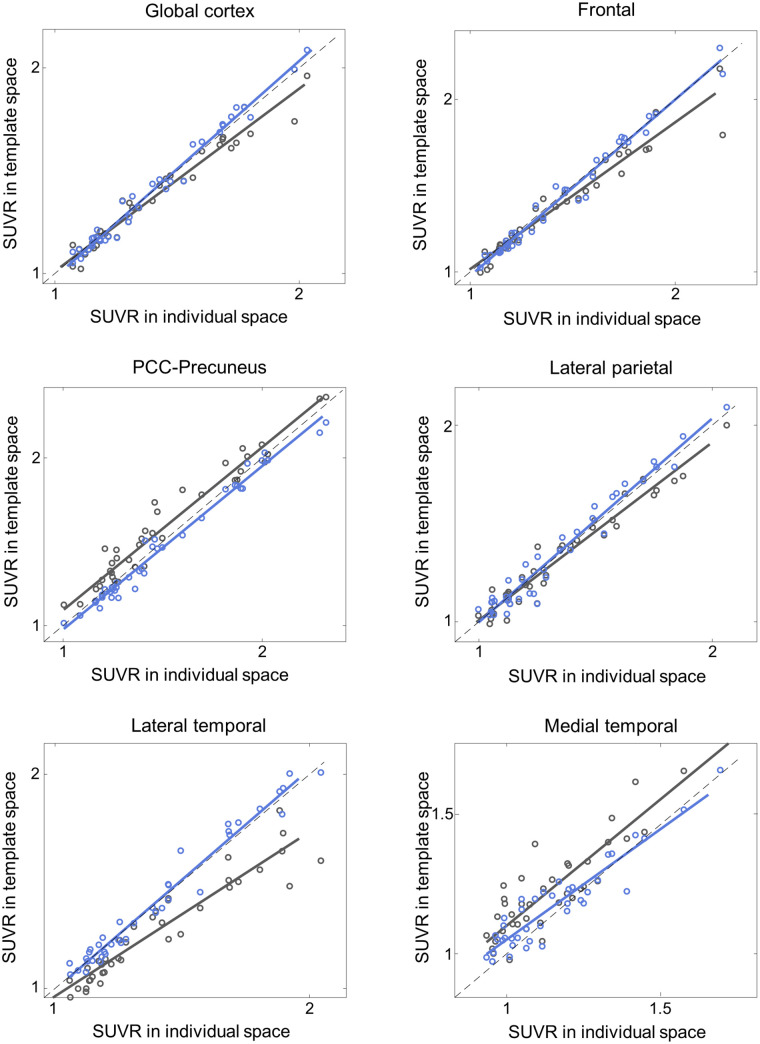
External validation: SUVR comparison in ^18^F-florbetapir (*n* = 39). *x*-axis represents ground truth SUVR estimated in individual space using FreeSurfer VOI, whereas *y*-axis represents SUVR estimated in template space using coregistered MRI and SPM (black symbols and lines) or proposed DNN (blue symbols and lines). PCC = posterior cingulate cortex.

Additionally, the proposed DNN method correlated better with the FreeSurfer approach than did SPM SN for all 3 tested radiotracers and most of the tested VOIs (Figs. 4–7; Tables 3–6). Furthermore, the proposed method yielded higher intraclass correlation coefficient results than did SPM in almost all comparisons (Tables 3–6). Moreover, the proposed method showed a lower bias in SUVR estimation in the Bland–Altman analysis (Supplemental Figs. 4–7). No remarkable differences were observed between the internal and external validation results. Although the ^18^F-florbetapir data were not used in the DNN training, the proposed method showed no performance degradation for the external ^18^F-florbetapir dataset. The results of separate analysis for amyloid-positive and -negative cases, which were divided by a global SUVR of 1.5, are summarized in Supplemental Tables 1–4.

**TABLE 3.** Internal Validation: Pearson Correlation and ICC Analysis for SUVR of Internal ^18^F-Florbetaben and ^18^F-Flutemetamol Dataset (*n* = 148) Relative to FreeSurfer Approach

	SPM				Proposed			
Parameter	Slope	*y-*intercept	*R* ^2^	ICC	Slope	*y-*intercept	*R* ^2^	ICC
Global	0.869	0.113	0.946	0.965	1.019	−0.016	0.986	0.992
Frontal	0.956	0.183	0.947	0.946	0.983	0.019	0.987	0.992
PCC–precuneus	0.877	0.158	0.950	0.921	0.998	0.026	0.981	0.993
Lateral parietal	0.734	0.267	0.910	0.970	0.936	0.092	0.977	0.988
Lateral temporal	0.853	0.173	0.957	0.865	1.008	0.003	0.987	0.987
Medial temporal	0.879	0.269	0.732	0.554	0.944	0.125	0.891	0.861

PCC = posterior cingulate cortex.

**TABLE 4.** External Validation: Pearson Correlation and ICC Analysis for SUVR of External ^18^F-Florbetaben Dataset (*n* = 30) Relative to FreeSurfer Approach

	SPM				Proposed			
Parameter	Slope	*y-*intercept	*R* ^2^	ICC	Slope	*y-*intercept	*R* ^2^	ICC
Global	0.853	0.167	0.979	0.972	1.003	−0.006	0.995	0.998
Frontal	0.836	0.181	0.983	0.966	0.970	0.010	0.995	0.994
PCC–precuneus	0.970	0.121	0.986	0.981	0.990	0.019	0.993	0.996
Lateral parietal	0.821	0.209	0.965	0.961	0.994	0.016	0.996	0.998
Lateral temporal	0.794	0.151	0.936	0.879	0.963	0.054	0.986	0.993
Medial temporal	0.972	0.134	0.898	0.800	0.990	0.062	0.931	0.927

PCC = posterior cingulate cortex.

**TABLE 5.** External Validation: Pearson Correlation and ICC Analysis for SUVR of External ^18^F-Flutemetamol (*n* = 67) Relative to FreeSurfer Approach

	SPM				Proposed			
Parameter	Slope	*y-*intercept	*R* ^2^	ICC	Slope	*y-*intercept	*R* ^2^	ICC
Global	0.907	0.104	0.979	0.977	1.033	−0.020	0.990	0.989
Frontal	0.893	0.117	0.976	0.975	1.025	−0.015	0.990	0.987
PCC–precuneus	0.978	0.150	0.978	0.945	1.024	−0.032	0.985	0.984
Lateral parietal	0.919	0.103	0.975	0.969	1.001	0.036	0.987	0.979
Lateral temporal	0.794	0.136	0.946	0.844	0.986	0.042	0.984	0.984
Medial temporal	0.943	0.206	0.857	0.758	0.921	0.149	0.926	0.931

PCC = posterior cingulate cortex.

**TABLE 6.** External Validation: Pearson Correlation and ICC Analysis for SUVR of External ^18^F-Florbetapir Dataset (*n* = 39) Relative to FreeSurfer Approach

	SPM				Proposed			
Parameter	Slope	*y-*intercept	*R* ^2^	ICC	Slope	*y-*intercept	*R* ^2^	ICC
Global	0.888	0.123	0.961	0.979	1.082	−0.071	0.982	0.985
Frontal	0.851	0.166	0.940	0.974	1.022	−0.045	0.980	0.989
PCC–precuneus	0.973	0.119	0.948	0.940	0.975	0.001	0.980	0.982
Lateral parietal	0.905	0.102	0.949	0.981	1.037	−0.039	0.958	0.978
Lateral temporal	0.768	0.196	0.892	0.854	1.029	−0.034	0.970	0.990
Medial temporal	0.977	0.128	0.798	0.742	0.855	0.199	0.864	0.936

PCC = posterior cingulate cortex.

The computation time required for PET SN using the proposed method was approximately 1 s. Conversely, SPM required more than 60 s for the batch operation, which included coregistration between PET and MRI, SN parameter estimation from MRI, and writing of the spatially normalized PET image. FreeSurfer required approximately 8 h for automatic MRI parcellation.

## DISCUSSION

In this study, we developed a fast amyloid brain PET SN method based on DNNs to overcome the limitations of existing approaches based on paired anatomic images or patient-specific templates (25*,*^26^*,*^32^). Furthermore, we assessed the correlation and measurement consistency between the proposed method and FreeSurfer-based SUVR quantification, which showed a strong correlation with the manual VOI approach (32). In terms of correlation and consistency with the FreeSurfer-based approach, the DNN-based PET SN method outperformed MRI-based PET SN conducted using the coregistration and SN routines of SPM, which is one of the most widely used pipelines for amyloid brain PET research.

The DNN model trained in this study allowed a robust SN of amyloid PET images without MRI. The superiority of the SN performance of the proposed method compared with that of SPM SN using MRI was most pronounced in cases with hydrocephalus, as shown in Figure 2 and Supplemental Figure 2. The DNN model trained using nearly 1,000 datasets with on-the-fly data augmentation was able to generate SN PET images that were morphologically consistent with the standard MRI template. Although the DNN model was trained using a Korean dataset, no performance difference was observed when it was applied to external datasets obtained from other countries. Accurate SN of the lesioned brain was also possible, as shown in Figure 3, without shrinkage of the lesion volume, which is frequently observed in conventional SN approaches (41). However, despite the use of MRI, SPM SN could not compensate for the large morphologic differences between the input images and the template. In the SN algorithm used in SPM, the images are deformed by the linear combination of 1,000 cosine transform bases, which allowed only a limited amount of image deformation.

A potential alternative approach to the proposed method is generating spatially normalized amyloid PET images directly from individual PET inputs using DNNs. This method is faster than the proposed method considering it directly conducts SN without generating explicit deformation fields. However, direct SN methods are more susceptible to the perturbation of input images because of noise. Therefore, it is difficult to ensure maintenance of regional count rate concentrations after the direct SN of brain PET images. However, the DNN model used in the proposed method does not directly provide the intensity of SN images. The intensities were calculated by interpolating neighbor voxel values using DNN-generated deformation fields, which reduced the risk of erroneous intensity mapping by the SN. In addition, the DNN model trained for deformation field generation using amyloid PET images can be used for transfer learning on other radiotracers with small datasets available. Our preliminary (unpublished data, June 2022) study on ^18^F-flortaucipir showed that the transfer learning allows for highly accurate quantification of ^18^F-flortaucipir brain PET using the proposed method.

The proposed fast and reliable deep-learning–based SN of amyloid PET images can potentially be used to improve interreader agreement on, and confidence in, amyloid PET interpretation. In our previous study (42), when visual amyloid PET interpretation was supported by a deep-learning model that directly estimated regional SUVR from input images (43), interreader agreement (Fleiss κ-coefficient) and the confidence score increased from 0.46 to 0.76 and from 1.27 to 1.66, respectively. The method proposed here requires a longer computation time for regional SUVR calculation than the direct end-to-end SUVR estimation, mainly because of the voxel-by-voxel multiplication of SN results and the predefined brain atlas. However, the reliability of the amyloid burden estimation based on the proposed method is higher, considering that the proposed method allows visual confirmation of SN results and exclusion of cases with erroneous SNs. Furthermore, accurate automatic quantification of amyloid burden can be used in longitudinal follow-up studies on patients with AD and mild cognitive impairment. Several dementia treatment drugs based on the amyloid hypothesis are now emerging, and amyloid PET scans are important for monitoring the efficacy of treatments. The proposed method will enable an objective measurement of drug-induced amyloid clearance without requiring additional 3-dimensional structural MRI.

## CONCLUSION

We evaluated a novel deep-learning–based SN method that allows quantitative analysis of amyloid brain PET images without structural MRI. The quantification results using the proposed method correlated strongly with MRI-parcellation–based quantification using FreeSurfer for all clinical amyloid radiotracers. Therefore, the proposed method will be useful for investigating AD and related brain disorders using amyloid PET scans.

## DISCLOSURE

This research was supported by the Seoul R&BD Program (BT200151) through the Seoul Business Agency (SBA) funded by the Seoul Metropolitan Government. No other potential conflict of interest relevant to this article was reported.

## Notes

KEY POINTS **QUESTION:** Is quantification of amyloid PET images without MRI feasible? **PERTINENT FINDINGS:** A method based on deep learning allowed fast and reliable amyloid PET SN and quantification without MRI. **IMPLICATIONS FOR PATIENT CARE:** The proposed method will be useful for interpreting amyloid PET scans in AD and related brain disorders.
